# Evaluation of the role of the rail sign and intracervical lakes in the management of patients with a high probability of placenta accreta spectrum

**DOI:** 10.1186/s12884-026-08766-2

**Published:** 2026-02-09

**Authors:** Ahmed M. Hussein, Mohamed M. Thabet, Rana M. Elbarmelgy, Rasha A. Elbarmelgy, Eric Jauniaux

**Affiliations:** 1 From the department of Obstetrics and Gynecology, Kasr Al Ainy School of Medicine, University of Cairo, Cairo, Egypt; 2 Institute for Women’s Health, Faculty of Population Health Sciences, University College London, 86-96 Chenies Mews, London, WC1E 6HX UK

**Keywords:** Placenta accreta spectrum, Placenta previa accreta, Complex caesarean section, Ultrasound imaging, Rail sign, Uterine cervix

## Abstract

**Background:**

Classical ultrasound signs of placenta accreta spectrum (PAS) at birth, including anomalies of the lower uterine segment (LUS) and uteroplacental and intraplacental circulations, are now well established. The purpose of this study was to evaluate the use of “intracervical lakes” and “the rail sign,” which are more recently described signs.

**Methods:**

We conducted a retrospective analysis of ultrasound imaging data and primary surgical outcomes of consecutive singleton pregnancies in patients with a history of at least one prior CD presenting with an anterior low-lying or placenta previa at 32–36 weeks. Ultrasound findings were recorded using a standardized protocol. The diagnosis of PAS was confirmed when one or more placental lobules could not be digitally separated from the uterine wall at delivery or during the gross examination of hysterectomy or partial myometrial resection (PMR) specimens, and confirmed by histopathology. All analyses were performed using a logistic regression.

**Results:**

Of the 227 patients in the cohort, 50 (22%) presented with intracervical lakes on transvaginal scan (TVS) and 97 (47.7%) with a rail sign on transabdominal sonography (TAS). A peripartum hysterectomy (PH) was performed in 116 cases (51%), and 97 patients were managed conservatively: 41 (18%) with PMR and LUS reconstruction, and 70 (31%) with a complex CD, with no intraoperative evidence of PAS. Placental lacunae were the strongest predictors of both PAS and PH, with a high lacunae score (3+) associated with odds ratios (ORs) of 320 (95% confidence interval (CI) 243,4231) for PAS and 9.00 (95% CI 3.01,26.9) for PH, respectively. Associations with PAS were also found for placental bulge (OR 8.24; 95% CI 2.54,26.8) and the rail sign (OR 3.01; 95% CI 1.04,8.67). Increased odds of PH were found for myometrial thinning of < 1 mm (OR 5.47; 95% CI 1.69,17.7) and the presence of intracervical lakes (OR 12.3; 95%CI 3.89,39.1).

**Conclusions:**

The presence of a rail sign was associated with an increased odds of PAS at birth, whereas the presence of intracervical lakes was associated with an increased odds of peripartum hysterectomy in patients with a history of CD who presented with a placenta previa.

**Trial registration:**

This study was prospectively registered. Ethical approval was obtained before the start of this study (Scientific and Research Ethical Committee approval at the University of Cairo, RSEC 021001). The study was conducted in accordance with the Declaration of Helsinki.

## Background

Ultrasound imaging has become an essential tool in the prenatal evaluation of patients at risk for placenta accreta spectrum (PAS). Early detection is essential for managing this condition, as it can lead to major maternal and fetal complications during delivery [1, 2]. Patients with a history of Cesarean delivery (CD) presenting with a low-lying placenta or placenta previa and ultrasound signs associated with PAS are at the highest risk of intra-operative massive hemorrhage and damage to the urinary tract, and their outcome is improved when managed electively by a multidisciplinary team (MDT) [3–5].

Accreta placentation was defined by pathologists in the 1930s as the abnormal attachment of part of the placenta to the uterine wall [5]. Imaging cannot formally diagnose PAS before birth, but it is highly effective in assessing the risk of PAS at delivery. Ultrasound imaging, in particular, has been successfully used to screen patients with a history of prior CDs presenting with a placenta partially or totally located under a lower uterine segment (LUS) cesarean scar [6–8]. When performed by skilled operators, pooled ultrasound performance for the prenatal detection of high-risk placenta previa accreta at birth ranges from 88 to 97% for sensitivity and 90–97% for specificity [9]. We also showed recently that preoperative ultrasound examination in patients with a high probability of PAS at birth contributes to the surgical planning and can support patient counselling and consent [10–14].

A recent study using a structured Delphi process informed by a systematic review confirmed the continued importance of seven of the 11 established standardised ultrasound signs, including the loss of the “clear zone”, myometrial thinning and bladder wall interruption, and the presence of a placental bulge, subplacental (uteroplacental) hypervascularity, placental lacunae, exophytic mass, and bridging vessels [8]. None of the new eight signs reached a predefined consensus threshold as ultrasound findings that increase the probability of PAS at birth, probably due to technical limitations in the availability of specific software on routine ultrasound equipment and limited prospective data on their use in patients with a high probability of PAS at birth.

The present study aimed to assess the roles of two more recently described signs of PAS that can be obtained with standard ultrasound equipment i.e. the presence of intracervical lakes [15] and of “the rail sign” [16] in the screening of patients at high risk of accreta placentation and in the preoperative evaluation of their surgical outcomes.

## Methods

We performed a retrospective analysis of ultrasound imaging data and primary surgical outcomes for consecutive singleton pregnancies in patients with a history of at least one prior CD presenting with an anterior low-lying placenta or placenta previa, collected prospectively between March 2019 and April 2025. All patients were referred for delivery by an expert specialist MDT at 32–36 weeks of gestation. Patients with multiple pregnancies or requiring emergency delivery were excluded from the prospective cohort.

All patients were managed by the same MDT according to local protocols, including for patients with PAS at birth, either peripartum hysterectomy (PH) or conservative surgical management, i.e., partial myometrial resection (PMR) of the accreta with reconstruction of the LUS when sufficient myometrial tissue was available after dissection of the utero-bladder interface.

A digital photographic protocol was used to capture images of the macroscopic features during the different phases of the surgery and gross examination of the hysterectomy specimens [17]. The diagnosis of PAS was confirmed when one or more placental lobules could not be digitally separated from the uterine wall at delivery or during the gross examination of the hysterectomy or PMR specimens. Samples were taken at the placenta-uterine interface of the abnormally attached cotyledons for histologic confirmation of diagnosis.

All patients underwent at least one detailed transabdominal sonography (TAS) and transvaginal scan (TVS) examination by the MDT, upon transfer to the department and within 48 h before CD, including color Doppler imaging (CDI) mapping of the placenta and uteroplacental interface (GE Voluson E10, GE Medical System, Zipf, Austria). The placenta was recorded as “low lying” when the edge was 0.5–2 cm from the internal os of the uterine cervix on TVS. When the placenta was < 0.5 cm from the internal os or completely covering it, it was defined as placenta previa (marginal or complete) [18]. Cervical length (CL), structural changes, and vascularity were evaluated in all cases by TVS.

Ultrasound findings were recorded prospectively using a standardized protocol, which included anomalies of the uterine contour and uteroplacental interface on grey-scale imaging (loss of clear zone, myometrial thinning, and placental bulge) and anomalies of the utero-placental and intraplacental circulations on CDI. The residual myometrial thickness (RMT) was measured at the thinnest site perpendicular to the long axis of the LUS, placing one calliper at the interface between the LUS and bladder walls and the other at the interface between the LUS wall and the placental bed or the amniotic cavity. The score proposed by Finberg and Williams was used to record intraplacental lacunae (0 = none; 1 + = 1–3; 2 + = 4–6; 3+=>6) [19]. The presence of feeder vessels to the lacunae was also recorded. All authors agreed upon all ultrasound descriptions and signs at the start of the prospective study.

**Fig. 1:**
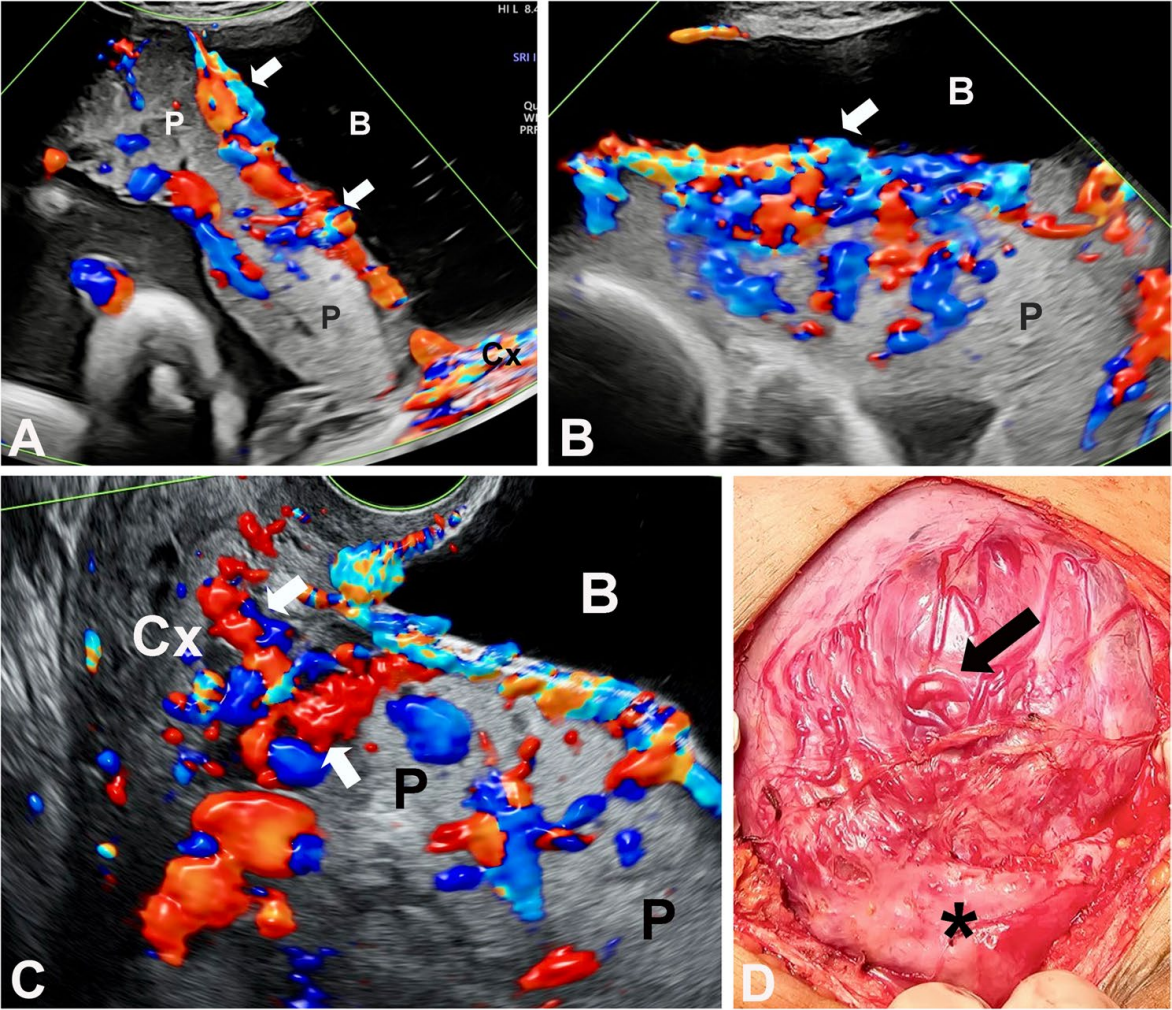
Case of a patient at 36 weeks with PAS at birth managed by peripartum hysterectomy. **A**: Longitudinal TAS CDI mapping of the LUS showing a placenta (P) previa partially covering the cervix (Cx) and showing uteroplacental (merged subplacental uterovesical) hypervascularity interconnected with bridging vessels (arrows) corresponding to a rail sign. Note the cervical increased vascularity; **B**: Transverse TAS CDI mapping of the same area as in A; **C**: TVS CDI mapping showing the increased vasculature at cervico-placental interface with large lakes filled with blood (arrows); **D**: Intraoperative view of the anterior LUS wall at laparotomy before dissection of the bladder (*) showing enlarged subserosal vessels running cranio-caudally and laterally in the anterior uterine serosa (arrow). P= Placenta; B= Bladder

**Fig. 2:**
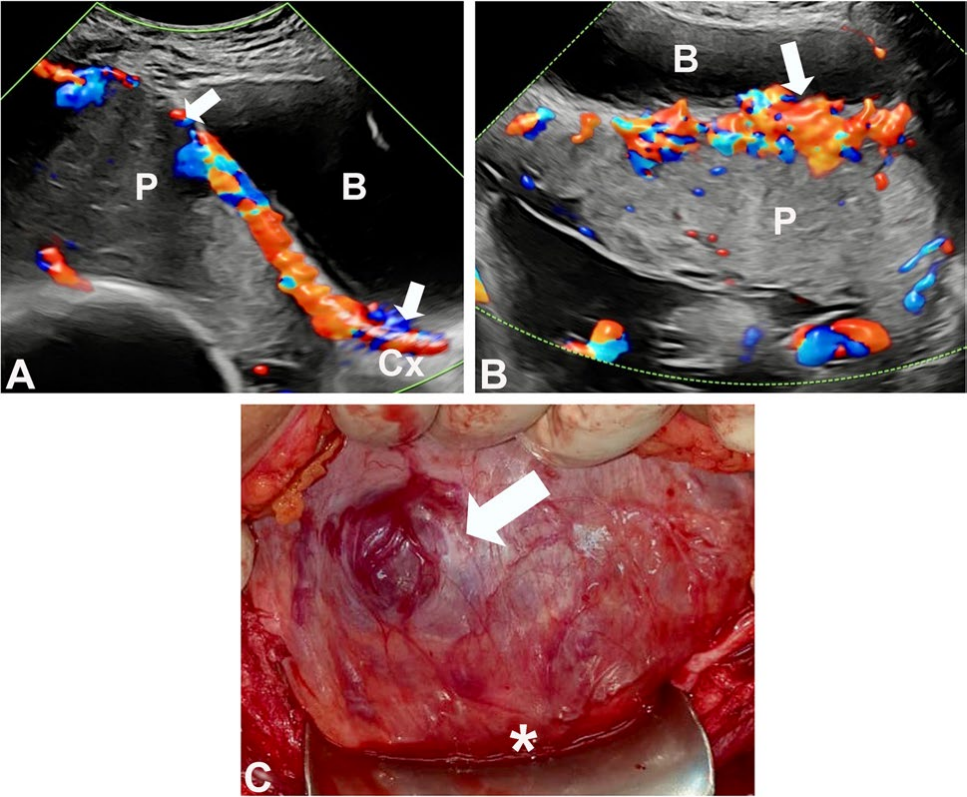
Case of a patient at 36 weeks with no evidence of PAS at birth. **A**: Longitudinal TAS CDI mapping of the LUS showing a placenta (P) previa partially covering the cervix (Cx) and showing increased uteroplacental vascularity along the utero-bladder interface (between arrows); **B**: Transverse TAS CDI mapping showed an area of focal merged subplacental uterovesical hypervascularity interconnected with bridging vessels (arrow) corresponding to a rail sign.; **C**: Intraoperative view of the anterior LUS wall at laparotomy before dissection of the bladder (*) showing a small area of enlarged subserosal vessels (arrow) corresponding anatomically to the ultrasound area (arrow) in B. P= Placenta; B= Bladder

The presence of intracervical lakes defined as tortuous hypervascularised anechoic spaces within the cervix on TVS [15] (Fig. 1) and of “the rail sign” described on as two parallel enlarged vessels over the uterovesical junction and bladder mucosa, with interconnecting bridging vessels perpendicular to both on TAS [16] (Figs. 1 and 2), were added to our standardized prospective protocol in 2020 and 2021, respectively.

### Ethics approval and consent to participate

Ethical committee approval was obtained before the start of this study (Scientific and Research Ethical Committee approval at the University of Cairo, RSEC 021001). All patients were informed and provided written consent for the use of ultrasound intraoperative images and videos for research and training purposes. Clinical data for this study were collected using a standard clinical audit protocol, and all data and images were fully anonymised before analysis.

### Statistical analyses

The outcomes of interest were: PAS confirmed at birth and management i.e. conservative management with uterine preservation or peripartum hysterectomy (PH). All analyses were performed using logistic regression. The association of each factor with each outcome was examined in a series of univariable analyses, and the joint association between the factors and the outcome was examined in a multivariable analysis. A backward selection procedure was used to choose the final regression model, omitting non-significant factors one at a time until all remaining factors were significant. SPSS V 28.0.1.1 (IBM Corp, Armonk, NY, USA) was used to analyse the data. The strength of the association between each factor and PAS was quantified using odds ratios (ORs) and 95% confidence intervals (CIs). A *p*-value < 0.05 was considered significant.

## Results

The cohort comprised 227 patients, including 50 (22%) who presented with intracervical lakes on TVS and 97 (47.7%) who had a rail sign on TAS. The median gestational age at delivery was 36 weeks and 2 days (range 34 weeks 1 day and 37 weeks 6 days), and the median number of previous CDs was 3 (range 1–7), with 117 patients having a history of ≥ 2 previous CDs.

There were 17 patients presenting with an anterior low-lying placenta (7.4%) and 210 with a placenta previa, including 28 (12.3%) described as anterior marginal (placental edge reaching the internal os of the cervix) and 182 with a placenta, mainly anterior covering the internal os. PAS was confirmed at birth in 128 patients (56%).

There were 116 (51%) patients who required a PH, 41 (18%) who were managed with PMR of the accreta area, followed by reconstruction of the LUS, and 70 (31%) patients who presented with an extended dehiscence of the LUS, with no intraoperative evidence of PAS that only required reconstruction of the LUS. In the subgroup of patients who had a PH, 27 (23%) underwent the procedure due to the lack of myometrial tissue above the cervix available for reconstruction after complete dissection of the LUS. The others had a primary PH for hemostasis during LUS dissection or PMR.

Table 1 presents the results of univariable analyses of the number of previous CDs, placental position, CL measurements, and the different ultrasound signs. No formal analysis was performed for loss of clear zone or bladder wall interruption, as all patients except one were found to have these signs. No patients presented with an “exophytic mass”. All other ultrasound signs examined were significantly associated with PAS.

**Table 1:** Univariable associations between clinical variables and ultrasound features with PAS

Variable	Category	PAS *n*/*N* (%)	Odds Ratio (95% CI)	*P*-value
Number of previous CD	-	-	1.24 (0.98, 1.57)	0.07
Placental position	Previa	121/210 (58%)	1	0.20
	Low-lying	7/17 (41%)	0.51 (0.19, 1.41)	
Cervical length ^(*)^	-	-	0.79 (0.61, 1.03)	0.08
Loss of clear zone	No	1/1 (100%)	-	**-**
	Yes	127/226 (56%)		
Myometrial thinning	> 2 mm	9/32 (28%)	1	**< 0.001**
(RMT)	1–2 mm	66/123 (54%)	2.95 (1.27, 6.91)	
	< 1 mm	53/72 (74%)	7.13 (2.81, 18.1)	
Bladder wall	No	128/227 (56%)	-	-
interruption	Yes	0/0		
Placental bulge	No	52/123 (42%)	1	**< 0.001**
	Yes	76/104 (73%)	3.71 (2.11, 6.50)	
Subplacental	Normal	18/84 (21%)	1	**< 0.001**
vascularity	Increased	110/143 (77%)	12.2 (6.38, 23.4)	
Placental lacunae	None	3/79 (4%)	1	**< 0.001**
	1+ / 2+	69/91 (76%)	79.5 (22.8, 277)	
	3+	56/57 (98%)	419 (143, 4000)	
Bridging vessels	No	77/165 (47%)	1	**< 0.001**
	Yes	51/62 (82%)	5.30 (2.58, 10.9)	
Intracervical lakes	No	85/177 (48%)	1	**< 0.001**
	Yes	43/50 (86%)	6.64 (2.83, 15.6)	
Rail sign	No	61/130 (47%)	1	**0.001**
	Yes	67/97 (69%)	2.53 (1.46, 4.38)	

(*) Odds ratio given for a 10-unit increase in cervical length, *RMT* Residual myometrial thickness, *CD* Cesarean delivery, *n/N* number cases of PAS with feature/total number of cases

The results for myometrial thinning indicated that PAS was least common among patients with an RMT >2 mm, with 28% having PAS. This contrasted with 74% of patients having PAS in the <1 mm category. A placental bulge was also found to be significantly (P < 0.001) associated with a higher risk of PAS (P < 0.001). The odds of PAS were 3.7 times higher for patients with a placental bulge compared to patients with no bulge. Placental lacunae were very strongly associated with PAS. PAS was rare in patients with no lacunae (4%), whereas almost all patients with 3 + lacunae (98%) had PAS. The presence of bridging vessels and increased subplacental (uterovesical) vascularity were both associated with an increased risk of PAS. The odds of PAS were 5 times higher in patients with bridging vessels than in those without this sign, and 12 times higher in patients with increased subplacental vascularity than in those with normal vascularity.

The presence of intracervical lakes and the rail sign were both significantly associated with PAS (*P* < 0.001 and *P* < 0.001, respectively). Patients with intracervical lakes had PAS odds 6.6 times higher than those with normal vascularity. Patients with a rail sign had 2.5 times the odds of PAS compared with those without a rail sign.

The multivariable analysis indicated that uteroplacental vascularity and placental lacunae were both significantly (*P* < 0.001) associated with PAS. Placental lacunae were found to be by far the strongest predictor of PAS. Patients with 3 + lacuna score (≥ 6 lacunae) had more than 300-fold higher odds of PAS than those with no lacunae (Table 2). The odds of PAS were 8 times higher in patients with a placental bulge than in those with no bulge, whilst the odds were 3 times higher in those with a rail sign.

**Table 2:** Multivariable model of the ultrasound features associated with PAS

Variable	Category	Odds Ratio (95% CI)	*P*-value
Placental bulge	No	1	**< 0.001**
	Yes	8.24 (2.54, 26.8)	
Placental lacunae	None	1	**< 0.001**
	1+ / 2+	175 (36.5, 840)	
	3+	320 (243, 4231)	
Rail sign	No	1	**0.04**
	Yes	3.01 (1.04, 8.67)	

The results of univariable analyses of the different clinical and ultrasound variables and surgical outcomes are presented in Table 3. For this analysis, patients who had a CD without additional surgical procedures and those who had a PMR with LUS reconstruction were combined in the conservative surgical management subgroup. Every previous CD was associated with a 45% increase in the odds of PH. Patients with an RMT <1 mm were at a greater risk of PH, with the odds being 6.6 times higher for this group than for women with >2 mm of thinning.

**Table 3:** Univariable associations between clinical variables and ultrasound features with surgical outcomes

Variable	Category	Peripartum hysterectomy *n*/*N* (%)	Odds Ratio ^(+)^ (95% CI)	*P*-value
Number of previous CD	-	-	1.45 (1.14, 1.84)	**0.003**
Placental position	Previa	111/210 (53%)	1	0.07
	Low-lying	5/17 (29%)	0.37 (0.13, 1.09)	
Cervical length ^(*)^	-	-	0.95 (0.74, 1.22)	0.72
Loss of clear zone	No	0/1 (0%)	-	**-**
	Yes	116/226 (51%)		
Myometrial thinning	> 2 mm	9/32 (28%)	1	**< 0.001**
	1–2 mm	55/123 (45%)	2.07 (0.88, 4.83)	
	< 1 mm	52/72 (72%)	6.64 (2.63, 16.8)	
Bladder wall	No	116/227 (51%)	-	-
interruption	Yes	0/0		
Placental bulge	No	52/123 (42%)	1	**0.004**
	Yes	64/104 (62%)	2.18 (1.28, 3.72)	
Subplacental	Normal	24/84 (29%)	1	**< 0.001**
vascularity	Increased	92/143 (64%)	4.51 (2.51, 8.09)	
Placental lacunae	None	22/79 (28%)	1	**< 0.001**
	1+ / 2+	45/91 (49%)	2.53 (1.34, 4.81)	
	3+	49/57 (86%)	15.8 (6.49, 38.8)	
Bridging vessels	No	71/165 (43%)	1	**< 0.001**
	Yes	45/62 (73%)	3.50 (1.85, 6.63)	
Intracervical lakes	No	71/177 (40%)	1	**< 0.001**
	Yes	45/50 (90%)	13.4 (5.09, 35.5)	
Rail sign	No	57/130 (44%)	1	**0.01**
	Yes	59/97 (61%)	1.99 (1.16, 3.40)	

(+) Odds Ratios represent the odds of CSH in each category relative to the odds in a baseline category

(*) Odds ratio given for a 10-unit increase in cervical length

The presence of a placental bulge and increased subplacental vascularity were both significantly (*P* < 0.004 and *P* < 0.001, respectively) associated with an increased chance of a PH. The odds for PH in patients with the increased subplacental vascularity were 4.5 times higher than for women without this feature, whilst the odds of PH were twice as likely for patients with a placental bulge. A higher score of placental lacunae was associated with an increased chance of a PH. The data indicated that 86% of the patients with a 3 + lacunae score underwent a PH, compared to only 28% of those with no placental lacunae. The odds of PH management for a 3 + lacunae score were 16 times higher than for those with no placental lacunae.

A PH was performed in 61% of patients with a rail sign, compared to 44% for those without. There was a very strong association between cervical vascularity and the management method. Intracervical lakes were associated with a higher risk of a PH. The odds of this outcome were 13 times higher in these patients compared to those with normal cervical vascularity.

Table 4 presents the results of the corresponding multivariable analysis after backward selection, retaining only variables associated with the outcome in the final model. Every previous CD was associated with a 55% increase in the odds of PH. The odds of PH in patients presenting with a placenta praevia were 6 times higher than for those with a low-lying placenta. A lower level of myometrial thinning was associated with a higher risk of PH. The odds of PH were 5 times higher when the RMT was <1 mm than when it was >2 mm. The odds of PH in patients with a high lacunae score (3+) were 9 times those in patients with no lacunae. The presence of intracervical lakes was associated with the highest risk of PH. The odds of PH in these patients were 12 times higher than in those with normal cervical vascularity.

**Table 4:** Multivariable model of the clinical factors and ultrasound features associated with surgical outcomes

Variable	Category	Odds Ratio (95% CI) ^(*)^	*P*-value
Number of CS	-	1.55 (1.14, 2.09)	**0.005**
Placental position	Previa	1	**0.01**
	Low-lying	0.16 (0.04, 0.65)	
Myometrial thinning	> 2 mm	1	**0.004**
	1–2 mm	1.90 (0.30, 5.63)	
	< 1 mm	5.47 (1.69, 17.7)	
Placental lacunae	None	1	**< 0.001**
	1+ / 2+	1.38 (0.66, 2.89)	
	3+	9.00 (3.01, 26.9)	
Intracervical lakes	No	1	**< 0.001**
	Yes	12.3 (3.89, 39.1)	

(*) Odds Ratios represents the odds of peripartum hysterectomy (PH) in each category relative to odds in a baseline category

## Discussion

Tabsh et al. [20] were the first to report a case of PAS on ultrasound examination in 1982, in a patient with a history of one previous CD presenting at 25 weeks of gestation with an anterior, low-lying placenta and absent subplacental sonolucency, with a thin (< 2 mm) LUS wall on TAS. They described their case as a placenta increta, but the histologic image included in their article showed placental villi simply apposed to the myometrium, suggesting that this was probably the first description of a low-lying placenta under a LUS dehiscence rather than that of an actual case of PAS [5].

Patients with a history of CD often present with imaging evidence of LUS dehiscence and remodeling, regardless of whether the placenta is previa [13]. In patients presenting with a low-lying placenta or placenta previa, the myometrial thinning on LUS will often allow the underlying placental tissue to herniate through the dehiscence, creating a bulge on prenatal imaging. In the present study, the finding of a placenta bulge was associated with a significantly (*P* < 0.001) increased (OR 8.24; 95%CI 2.54,26.8) probability of PAS at birth, however, anomalies of the uterine contour and uteroplacental interface [21], including the loss of the clear zone, myometrial thinning, bladder wall interruption and placental bulging arise as a result of LUS scarring and occur, independently of accreta placentation.

In contrast, anomalies of uteroplacental circulation have consistently been linked to PAS at birth [8, 21, 22]. Data on the ongoing development of uteroplacental circulation in pregnancies implanted in the scar of a previous cesarean are limited to case reports and small cohort studies [23–25]. However, those pregnancies complicated by PAS often present with increased subplacental vascularization and lacunae from the end of the first trimester [2, 25, 26]. These changes are the consequence of placentation in a cesarean scar defect, where the standard uterine structure has been permanently replaced by a thin layer of scar tissue [5, 27], allowing the villous tissue to develop next and extravillous trophoblastic cells to reach the large arterial vessels of the uterine periphery [28–30]. As a result, the intervillous space of the corresponding lobule is supplied by radial or arcuate arteries with abnormally high velocity flow, distorting the placental anatomy and the uteroplacental interface, which progressively leads to abnormal attachment [31]. Placental lacunae can be quantified, but the definition of what constitutes subplacental “hypervascularity” remains elusive [8]. This could explain why the presence of cervical lacunae [15], which is part of the increased vascularisation of LUS in placenta previa accreta, was not associated with PAS in our multivariable analysis. The etiopathology of the rail sign remains uncertain [16]. It is probably a consequence of utero-bladder interface remodeling by CD scar tissue and adhesions, similar to that associated with bridging vessels and bladder wall interruption, and which is independent of PAS (Fig. 2).

Patients with a history of multiple CDs and placenta previa often present with major LUS remodeling and changes in the uteroplacental circulation [10–13, 32]. Major disruptions of LUS architecture, such as those associated with placental bulge, are strongly associated with intra-partum hemorrhage and the need for hemostatic PH, independently of the PAS [10, 11]. In the present study, placenta previa, myometrial thinning with an RMT of <1 mm, a high lacunae score, and intracervical lakes were all associated with increased odds of PH (Table 4).

TVS is essential for evaluating LUS anatomy, assessing cervical length, and accurately determining the position of the lower placental edge in a low-lying placenta and placenta previa [33]. TVS also improved prenatal screening accuracy for patients with PAS at birth among less-experienced operators [34]. There is limited data on the use of TVS in the preoperative evaluation of the level of surgical complexity in patients with a high probability of PAS at birth [35, 36]. Aryananda et al. [37, 38], using two-dimensional (2D) and three-dimensional (3D) CDI, recently proposed a TVS ultrasound grading system to evaluate the degree of cervical vascularity. The need for PH is associated with myometrial thinning of the distal part of the LUS and increased cervical vascularity on TVS [35], whereas the combination of intracervical hypervascularity > 50% and bladder wall remodelling has the highest predictive probability for PH [36]. These findings should now be used to develop study protocols to further assess the role of preoperative evaluation of patients at risk of complex CD.

The main strength of our study is that the data were obtained for a large continuous cohort of patients managed by the same MDT, using a standardised protocol for ultrasound examination of patients at high risk of PAS at birth. In all cases, the intraoperative findings were digitally recorded (photos and video clips), and detailed histopathologic examination of samples of abnormally attached placental lobules in cases of PH and PMR allowed us to accurately confirm the diagnosis of PAS and identify non-PAS cases that may present with major uterine remodelling without any abnormal placental attachment. The primary limitation of the current study lies in its retrospective design. Although the established ultrasound signs were recorded prospectively from the beginning of the study, the ultrasound descriptions of intraplacental lakes and the rail sign were published only one [15] and two [16] years later, respectively, and had to be examined retrospectively. The corresponding ultrasound images were jointly reviewed by the authors, and their classification was based on consensus using the criteria published in the literature. Another limitation is that most patients were referred from primary care centres with access to only basic ultrasound equipment. As a result, we could not evaluate changes in the placental position or the ultrasound appearance of signs associated with PAS, particularly those that require CDI and TVS.

## Conclusions

We found that the presence of the rail sign was associated with PAS at birth. The presence of intracervical lakes did not contribute significantly to the prenatal screening of PAS but was associated with a higher rate of PH. The presence of intracervical lakes, particularly when associated with placental lakes and placental bulging, is an essential parameter in the preoperative evaluation of the complexity of the surgical procedure.

## Data Availability

The datasets used and detailed statistical analysis are available from the corresponding author on reasonable request.
